# Factors associated with onward SARS-CoV-2 transmission among household and dormitory contacts of cases in Brunei Darussalam, August 2021 to February 2022: A retrospective cohort study

**DOI:** 10.1016/j.ijregi.2026.100888

**Published:** 2026-04-01

**Authors:** Liling Chaw, Kai Shing Koh, Muhammad Ali Rosledzana, Alex R. Cook, Justin Wong

**Affiliations:** 1 PAPRSB Institute of Health Sciences, Universiti Brunei Darussalam, Gadong, Brunei Darussalam; 2 Brunei Centre for Disease Control and Prevention, Ministry of Health, Bandar Seri Begawan, Brunei Darussalam; 3 Saw Swee Hock School of Public Health, National University of Singapore and National University Health System, Singapore, Singapore

**Keywords:** COVID-19, Contact tracing, Transmission, Setting-specific, Brunei

## Abstract

**Objectives:**

We aimed to determine the factors associated with contacts testing positive within a typical household and worker dormitory clusters during the COVID-19 Delta and Omicron waves.

**Methods:**

We performed retrospective analyses of national COVID-19 cases and contacts data sets in Brunei Darussalam, from August 2021 to mid-February 2022.

**Results:**

We identified 22,540 laboratory-confirmed cases, whereby 50.9% and 25.9% were from household and dormitory clusters, respectively. The median (interquartile range) serial interval for households (3 days [2–5]) was shorter than that of dormitories (5 days [3–9], *P* <0.001). Merging with the contact data set provided 14,289 household (and 10,900 dormitory) contacts from 2817 (and 1071) index cases. The overall secondary attack rate was similar for household (31.7% [95% confidence interval (CI): 31.0-32.5]) and dormitory contacts (27.4% [95% CI: 26.6-28.3]). For all contacts, the adjusted odds ratios (aORs) increased with decreasing vaccine doses acquired at exposure and if their index cases were symptomatic and infected during the Omicron period. The aORs were higher among older household contacts (aged >40 years) and immediate family members of their index cases. In contrast, the aORs for dormitory contacts were higher among male index cases.

**Conclusion:**

Our findings suggest a significant role of housing composition in within-household transmission dynamics.

## Highlights

• We reported COVID-19 transmission during Delta and Omicron variant waves in Brunei.
• Analysis was done for close contacts in household and worker dormitory cluster types.
• The serial interval was shorter in households than dormitories, but their secondary attack rates were similar.
• Higher adjusted odds ratios were found among older and immediate family members in households.
• Highlight more studies on the role of housing composition in transmission dynamics.

## Introduction

Before the availability of vaccines and therapeutic interventions, the global spread of SARS-CoV-2 had severely tested the preparedness and response capacities of national governments. Because countries are now learning from the 2020 SARS-CoV-2 pandemic to prepare for future ones, it is crucial to evaluate the response efforts that have been introduced to control the spread of SARS-CoV-2. Brunei Darussalam (population 440,000) is a small, well-connected country in Southeast Asia [1]. A typical Bruneian household is characterized by a multi-generation family structure, and group gatherings generally center around celebratory or religious events [2]. Foreign workers comprise about 27% of the country’s labor force, with 78% of them employed in construction, manufacturing, service, and retail trade [3]. Being a small country with closely linked local and migrant communities, Brunei is particularly vulnerable to outbreaks with potential widespread community transmission [4]. Notably, its small size also permits a degree of standardization of response to outbreaks that may be challenging in larger countries, such as extensive contact tracing.

The first SARS-CoV-2 case in Brunei was detected in March 2020, where all subsequent cases could be traced to an importation event and had clear epidemiologic links [5]. Using a test-trace-isolate approach, this first COVID-19 wave was generally contained successfully without widespread community transmission. However, in early August 2021, a new wave started wherein the first local community case had no clear epidemiological link. The high transmissibility of the Delta and then Omicron variants resulted in transmission occurring in settings other than within a typical household or mass gathering event. This led to the failure of the containment strategy that was previously successful, a scenario similar to other countries that had theretofore implemented successful measures against COVID-19 [[6], [7], [8]].

Many countries have previously reported associated factors of onward transmission, particularly, during the Alpha and Delta waves, and within typical household settings [6,[9], [10], [11], [12]]. There were a few reports on non-typical living settings, such as dormitories [7]. Brunei is one of the few countries where active contact tracing was continued up until the early part of the Omicron wave. Using an already-collected contact tracing data set, it is possible to determine case cluster types and identify their household contacts. Thus, we report the transmission characteristics of cases and contacts identified during the Delta and early Omicron wave, with emphasis on the two major cluster types in Brunei (household and worker dormitory). We also assessed the risk factors associated with onward secondary transmission at each cluster type. Our study findings could be helpful in the preparation, planning, and response efforts for future respiratory virus outbreaks and contribute to the existing literature on parameterizing disease transmission models.

## Methods

### Data collection

A line list of confirmed SARS-CoV-2 cases and their contacts was obtained from Brunei’s Ministry of Health, collected as part of their COVID-19 outbreak response activities [2]. A confirmed SARS-CoV-2 case was one who tested positive for SARS-CoV-2 through real-time reverse transcription–polymerase chain reaction testing on a nasopharyngeal swab specimen. Laboratory-confirmed cases were automatically reported to the outbreak response team to initiate the contact tracing process. A close contact was defined as any person living in the same household or someone within 1 m of a confirmed case in an enclosed space for ≥15 minutes. Laboratory testing for cases and contacts was conducted irrespective of symptom presentation. S1 text details the case and contact identification and management process, and Figure S1 shows the timeline of implemented public health measures from early March 2021 to April 2022.

The data set was compiled from epidemiologic week 30, 2021 to week 7, 2022 (July 25, 2021 to February 19, 2022), covering the whole Delta period and the early part of the Omicron wave (thereafter referred to as the Omicron period). The end date was selected because it was the last day of active contact tracing due to operational constraints in handling exponentially increasing case counts (Figure S2). Based on available SARS-CoV-2 sequencing results (Figure S3), we defined the Delta period as from epidemiologic weeks 30-51, 2021 (21 weeks; July 25 to December 25, 2021) and the Omicron period as from weeks 4-7, 2022 (4 weeks; January 23 to February 19, 2022). The intervening liminal period (i.e. week 52, 2021 to week 3, 2022; December 26 2021 to January 22, 2022) was removed from the analysis. It contained relatively few case counts (a total of 506 cases and 90 clusters).

The following case information was collected: socio-demographics, type of case (local or imported), disposition (death or recovery), and timings (dates of symptom onset, swab collection, and COVID-19 vaccine administration). Deaths were defined as COVID-19–related if it was due to respiratory failure and/or complications from pneumonia. Although the contact information collected includes socio-demographics, the case number of the case who first triggered the contact investigation, if they lived in a household as the index, the relationship between the contact and index, their swab test results, and timings (dates of COVID-19 vaccine administration, quarantine start, and last exposure to the index case).

### Definitions used

A cluster is where two or more cases were defined as epidemiologically linked if all three conditions were met: (i) had the same residential address, (ii) the difference in their symptom onset and/or swab collection dates was between −5 and +14 days, and (iii) based on observations during contact tracing investigations. Clusters that have occurred within a typical household or an accommodation for workers were defined as household and dormitory cluster types, respectively. The serial interval (SI) was calculated by identifying possible infector–infectee pairs among symptomatic cases from each cluster. To ensure true pairs were included in the SI calculation, we included pairs with a symptom date difference of between 1 and 15 days [13]. S1 text details the cluster identification and SI pair determination methods.

An index case was defined as one with the earliest symptom onset or swab collection date within the respective cluster. If multiple cases were swabbed on the same day, the case with the earliest symptom onset date and/or with the smallest case registration number was assigned as the index case. An asymptomatic case was defined as one who reported none of the following symptoms at the time of interview or swab collection (thereafter referred to as “at diagnosis”): fever; cough; sore throat, runny nose, sneeze, shiver, shortness of breath, headache, body and joint pain, body weakness; nausea; vomiting; and loss of taste, smell, and appetite. Cases were considered as vaccinated with one, two, or three doses when they had received their first, second, or third dose, respectively, at least 14 days before their symptom onset or swab collection date. Vaccination status of contacts was defined similarly, based on the difference between their most recent vaccination date and either their last exposure date to the index case or quarantine start date.

A household member was defined as one living in the same residential address as the case. Four categories were used to determine the relationship between a case and contact: (i) immediate family as one’s smallest family unit (including parent, sibling, spouse, son, daughter); (ii) extended family as one’s other family members (including grandparents, grandchildren, cousin, nephew/niece, relatives, spouse’s sibling, and housekeeper); (iii) work/school as one’s work colleague or school mate (including classmate, teacher, work colleague); and (iv) other, classified as any other relationships and health care worker.

### Statistical analysis

Epidemiologic and clinical characteristics were reported for the overall case population and for those belonging to household and dormitory cluster types. We first visualized and compared differences in the SI distribution between both cluster types [13] by drawing histograms and cumulative density function plots. Anderson–Darling k-sample and Mann–Whitney tests were conducted to compare their SI distribution and median values, respectively.

Next, a risk factor analysis was conducted where the case and contact datasets were based on the index case number recorded in the contact data set. After merging and initial exclusion (Figure S4), we extracted contacts who reported as living in the same household as their household or dormitory of the index case (thereafter referred to as household and dormitory contacts). We also calculated the secondary attack rate (SAR), defined as the percentage of contacts who have tested positive for the SARS-CoV-2 virus, and with 95% confidence intervals (CIs) estimated using the Agresti–Coull method. Contacts with missing test results were considered as negative; this was chosen as a conservative approach, such that any findings could be biased toward the null.

Generalized estimating equation (GEE) models were used to determine any factors associated with a positive test result among household contacts; the method allows us to account for clustering within the household of the index case. The explanatory variables evaluated included age, gender, and vaccination status for cases and contacts. For cases, their symptom status and wave period at diagnosis were included. We also included the contacts’ relationship with the index case variable. These variables were known to be associated with onward respiratory virus transmission [6,9,11,12] and included in the univariate and multivariable GEE analyses. We used binomial GEE, with a logit link function, and an independence correlation structure, chosen based on the Quasi-likelihood Information Criterion (QIC) [14]. We analyzed household and dormitory contacts separately, using the exact cluster type definition from the case data set. R (ver. 4.2.3) [15] was used in all analyses, including gee and geepack [16] packages. A *P* <0.05 was considered statistically significant.

## Results

### Case characteristics

**Figure 1:**
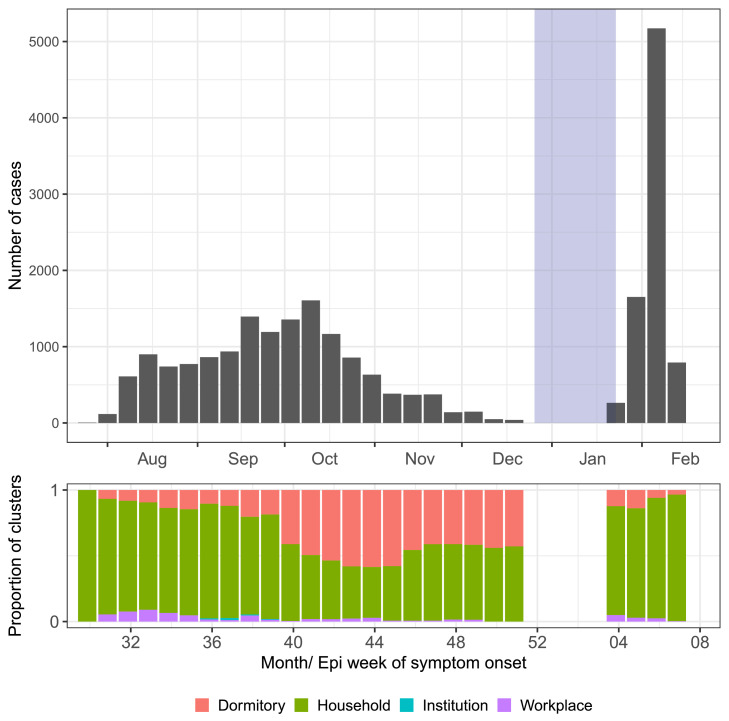
The epidemic curve of SARS-CoV-2 cases for whom an active contact tracing investigation was conducted in Brunei Darussalam, August 2021 to February 2022. The area shaded in blue shows the intervening liminal period between the Delta (left) and Omicron (right) periods. The stacked bar plot shows the weekly changes in the cluster proportion over time. Note that there is a discrepancy in the case counts in the second week of February 2022 because contact tracing was not conducted for all cases due to operational constraints. The whole epidemic curve is shown in Figure S2.

**Figure 2:**
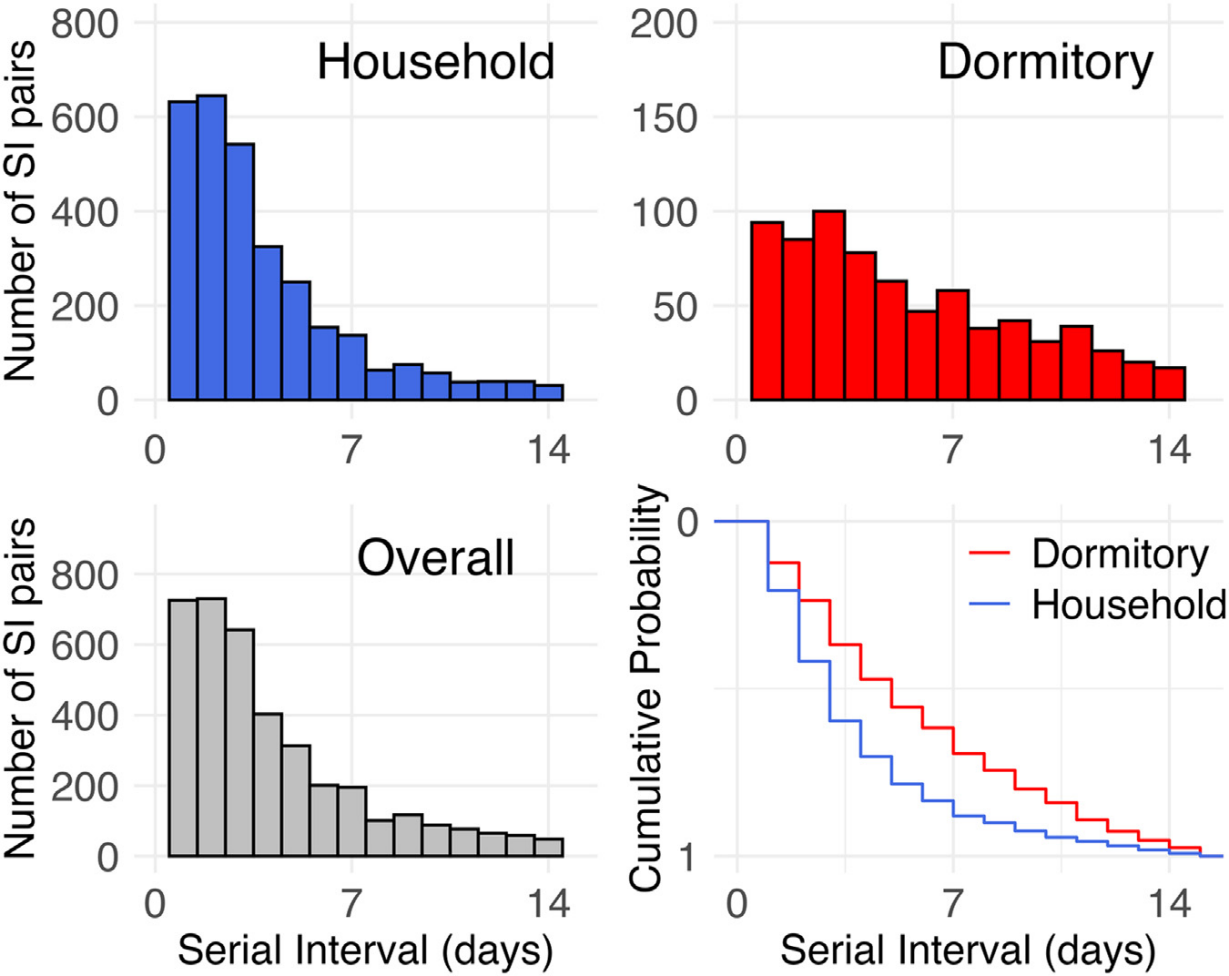
The Serial Interval (SI) of SARS-CoV-2 cases in Brunei Darussalam, August 2021 to February 2022. The SI distribution for household pairs (top left), worker dormitory pairs (top right), and overall (bottom left). Cumulative distribution plot showing the SI distribution of SI pairs by cluster type (bottom right).

There were 22,540 laboratory-confirmed SARS-CoV-2 cases, of which 14,659 (63.6%) and 7881 (34.2%) were identified during the Delta and Omicron periods, respectively (Figure 1). About half (50.9%, n = 11,468) and a quarter (25.9%, n = 5836) of all cases were classified as from household and dormitory cluster types, respectively, (Figure 2, Table 1). The median age was 26 years (interquartile range [IQR]: 12-41) and 36 years (IQR: 30-43) for household and dormitory cluster types, respectively. Males were disproportionately much higher for the dormitory cluster (Table 1). Most were locally transmitted cases (>97%). At the time of swab collection, 33.1% (n = 3794) of household and 61.6% (n = 3595) of dormitory cases were asymptomatic. The proportion of unvaccinated cases was highest during the Delta period, whereas that of cases with two or more doses was highest during the Omicron period.

SI, serial interval.

**Table 1:** Sociodemographic and clinical characteristics of SARS-CoV-2 cases in Brunei Darussalam for overall and by household and dormitory cluster type, August 2021 to February 2022.

Variables		a Total (%)	Household			Dormitory		
Case counts			Overall	Delta	Omicron	Overall	Delta	Omicron
		22,540 (100)	11,468 (51)	6621 (58)	4847 (42)	5836 (26)	5096 (87)	740 (13)
Age (in years)	Median (interquartile range)	32 (21-42)	26 (12-41)	26 (12-41)	26 (12-40)	36 (30-43)	36 (29-42)	38 (31-44)
	Range	0-94	0-94	0-94	0-90	0-70	0-70	1-63
Age group (in years)	0-9	2596 (12)	2363 (21)	1345 (20)	1018 (21)	16 (0)	12 (0)	4 (0)
	10-19	2642 (12)	2145 (19)	1273 (19)	872 (18)	24 (0)	17 (0)	7 (1)
	20-29	4644 (21)	1996 (17)	1097 (17)	899 (19)	1392 (24)	1261 (25)	131 (8)
	30-39	5655 (25)	1888 (17)	1102 (17)	786 (16)	2330 (40)	2059 (40)	271 (37)
	40-49	4178 (19)	1456 (13)	849 (13)	607 (13)	1592 (27)	1327 (26)	265 (36)
	50-59	1850 (8)	885 (8)	537 (8)	348 (7)	453 (8)	397 (8)	56 (8)
	60-69	673 (3)	514 (5)	299 (5)	215 (4)	26 (1)	22 (0)	4 (1)
	70+	287 (1)	212 (2)	117 (2)	95 (2)	1 (0)	1 (0)	0 (0)
Gender	Female	7737 (34)	5719 (50)	3217 (49)	2502 (52)	336 (6)	245 (5)	91 (12)
	Male	14794 (67)	5747 (50)	3403 (51)	2344 (48)	5498 (94)	4851 (95)	647 (88)
Symptoms at diagnosis	None	9719 (43)	3794 (33)	2773 (42)	1021 (21)	3595 (62)	3235 (64)	360 (49)
	Fever	6912 (31)	4280 (37)	2127 (32)	2153 (44)	1142 (20)	972 (19)	170 (23)
	Cough	7120 (32)	4377 (38)	2188 (33)	2189 (45)	1159 (20)	962 (19)	197 (27)
	Sore throat	2291 (10)	1367 (12)	455 (7)	912 (19)	214 (4)	150 (3)	64 (9)
	Runny nose	4748 (21)	3010 (26)	1416 (21)	1594 (33)	656 (11)	516 (10)	140 (19)
	Headache	1307 (6)	659 (6)	336 (5)	323 (7)	287 (5)	248 (5)	39 (5)
Vaccination status at infection	None	10167 (45)	6313 (55)	5075 (77)	1238 (26)	2019 (34)	2008 (39)	11 (2)
	1 dose	2627 (12)	843 (7)	815 (12)	28 (1)	1280 (22)	1278 (25)	2 (0)
	2 doses	7136 (32)	3072 (27)	728 (11)	2344 (48)	2200 (38)	1806 (36)	394 (53)
	3 doses	2610 (11)	1240 (11)	3 (0)	1237 (26)	337 (6)	4 (0)	333 (45)
COVID-19–related deaths	Yes	53 (0.2)	39 (0.3)	39 (0.6)	0 (0)	3 (0.1)	3 (0.1)	0 (0)
	No	33 (0.2)	17 (0.2)	17 (0.3)	0 (0)	0 (0)	0 (0)	0 (0)
	Unknown	9 (0)	5 (0)	5 (0)	0 (0)	3 (0.1)	3 (0.1)	0 (0)

a The total column shows the characteristics for the whole COVID-19 case group, including those outside of the household and dormitory cluster types.

A total of 95 deaths occurred during the Delta period, of which 53 (55.8%) were attributed to COVID-19. This gives a case fatality rate of 0.6% (95% CI 0.5-0.8) for the whole Delta period. Among these deaths, 39 (41.1%) and three (3.2%) were from the household and dormitory cluster types, respectively. No deaths were recorded in the Omicron period because this study only covers the first 4 weeks of the Omicron wave.

### Serial Interval (SI)

A total of 3809 transmission pairs were identified from household (80.1%, n = 3052) and dormitory (19.9%, n = 757) cluster types. Although roughly equal proportions were identified for both periods (Delta 1477 pairs [48.4%] and Omicron 1575 pairs [51.6%]), more than four-fifths of all dormitory pairs occurred during the Delta period (Delta 622 pairs [82.2%] and Omicron 135 pairs [17.8%]).

The overall median (IQR) SI for the household (3 days [[2], [3], [4], [5]]) was shorter than that of the dormitory clusters (5 days [[3], [4], [5], [6], [7], [8], [9]], Mann–Whitney *P* <0.001). Significant differences were also observed in the SI distribution of both cluster types (Anderson–Darling k-sample *P* <0.001) (Figure 2). Within 3 days from exposure to an infected index case, about 60% and 40% of infectees would develop symptoms in household and dormitory cluster types, respectively.

### Contact characteristics

A total of 70,514 contacts were recorded during the study period, of whom 46,074 (57.7%) remained after applying the initial exclusion criteria (Figure S4). We further excluded those who were not living with their index case, resulting in 14,389 (45.5%) household and 10,900 dormitory (75.5%) contacts. Household contacts were identified from 2817 index cases, with a median of four contacts per index case (IQR: 2-7) (Table 2). Although dormitory contacts were identified from 1071 index cases, with a median of five contacts per index case (IQR: 2-12).

**Table 2:** Demographic characteristics for contacts of SARS-CoV-2 cases who live with their household or dormitory index case in Brunei Darussalam, August 2021 to February 2022.

		Total	Household contacts			Dormitory contacts		
			Overall	Delta	Omicron	Overall	Delta	Omicron
		n (%)	n (%)	n (%)	n (%)	n (%)	n (%)	n (%)
Total contacts		46,074	14,389 (57)	12,074 (84)	2315 (16)	10,900 (43)	10,365 (95)	535 (5)
Index case counts		3888	2817 (72)	2284 (81)	533 (19)	1071 (28)	1014 (95)	57 (5)
Contact counts per index case	Median (IQR)	4 (2-8)	4 (2-7)	4 (2-7)	4 (2-6)	5 (2-12)	5 (3-12)	3 (2-7)
	Range	1-153	1-32	1-32	1-30	1-153	1-153	1-84
Age (in years)	Median (IQR)	32 (22-42)	26 (13-40)	26 (13-40)	27 (14-42)	36 (30-43)	37 (30-43)	38 (32-44)
	Range	0-96	0-94	0-94	0-90	0-78	0-78	8-72
Age group (in years)	0-9	3991 (9)	2784 (19)	2351 (20)	433 (19)	31 (0)	28 (0)	3 (1)
	10-19	5821 (13)	2426 (17)	2021 (17)	405 (18)	43 (0)	37 (0)	6 (1)
	20-29	10,016 (22)	2920 (20)	2465 (20)	455 (20)	2323 (21)	2231 (22)	92 (17)
	30-39	11,738 (26)	2550 (18)	2175 (18)	375 (16)	4355 (40)	4169 (41)	186 (35)
	40-49	8360 (18)	1645 (11)	1376 (11)	269 (12)	3033 (28)	2846 (28)	187 (35)
	50-59	4001 (9)	981 (7)	797 (7)	184 (8)	1035 (10)	979 (9)	56 (11)
	60-69	1490 (3)	771 (5)	630 (5)	141 (6)	74 (1)	70 (1)	4 (1)
	70+	574 (1)	312 (2)	259 (2)	53 (2)	6 (0)	5 (0)	1 (0)
Gender	Female	15,947 (35)	6753 (47)	5550 (46)	1203 (52)	629 (6)	538 (5)	91 (19)
	Male	30,119 (65)	7636 (53)	6524 (54)	1112 (48)	10,271 (94)	9827 (95)	444 (83)
Vaccination status at exposure	Unvaccinated	21,241 (46)	7873 (55)	7357 (61)	533 (23)	3617 (29)	3207 (31)	19 (4)
	1 dose	8957 (20)	2344 (16)	2323 (19)	7 (0)	2602 (24)	2579 (25)	1 (0)
	2 doses	13,927 (30)	3488 (24)	2342 (19)	1133 (49)	4946 (45)	4568 (44)	338 (63)
	3 doses	1949 (4)	684 (5)	52 (1)	642 (28)	185 (2)	11 (0)	177 (33)
Relationship type	Immediate family	6935 (15)	6375 (44)	5132 (43)	1243 (54)	119 (1)	100 (1)	19 (4)
	Extended family	7109 (15)	5813 (40)	5112 (42)	701 (30)	68 (1)	17 (1)	17 (3)
	Work/School	16,738 (36)	400 (3)	338 (3)	62 (3)	8146 (75)	7752 (75)	394 (74)
	Other	7344 (16)	1801 (13)	1492 (12)	309 (13)	2567 (24)	2462 (28)	105 (20)
Swab results	Negative	31,437 (68)	8934 (62)	8019 (66)	915 (40)	7231 (66)	7043 (68)	188 (35)
	Positive	9559 (21)	4566 (32)	3526 (29)	1040 (45)	2992 (27)	2749 (27)	243 (45)
	Missing	5078 (11)	889 (6)	529 (4)	360 (16)	677 (6)	573 (6)	104 (20)

IQR, interquartile range.

The overall SAR was higher for the household contacts (31.7% [95% CI: 31.0-32.5]) than for the dormitory (27.4% [95% CI: 26.6-28.3]). This difference was driven by contacts detected during the Delta period (Delta SAR household: 29.2% [95% CI: 28.4-30.0] vs SAR dormitory: 26.5% [95% CI: 25.7-27.4]) because the SAR for both cluster types during the Omicron period were similar (Omicron SAR household: 44.9% [95% CI: 42.9-47.0] vs SAR dormitory: 45.4% [95% CI: 41.2-49.7]). Contacts with missing test results (11% of all included contacts) (Table 2) were assumed to have tested negative. Although equal proportions of such contacts were observed in both household and dormitory groups (6%) (Table 2), the proportion of contacts with missing test results was higher during the Delta (70.4%) than the Omicron period (29.6%).

### Risk factors of transmission within cluster types

Three common findings were observed for both cluster types (Table 3). First, compared with unvaccinated contacts, household contacts who were vaccinated with one, two, or three doses had 51% (adjusted odds ratio [aOR]: 0.49 [95% CI: 0.43-0.57]), 63% (aOR: 0.37 [95% CI: 0.32-0.43]), and 70% (aOR: 0.30 [95% CI: 0.22-0.39]) lower odds of testing positive for SARS-CoV-2 virus, respectively. In a similar trend, dormitory contacts who were vaccinated with one, two, or three doses had 33% (aOR: 0.67 [95% CI: 0.55-0.81]), 45% (aOR: 0.55 [95% CI: 0.42-0.67]), and 63% (aOR: 0.34 [95% CI: 0.20-0.64]) lower odds of testing positive for SARS-CoV-2 virus, respectively. When repeating the analysis for each variant separately, this trend was still observed during the Delta (for one and two doses) and Omicron periods (for two and three doses), albeit with a wide 95% CI for dormitory contacts during the Omicron period (S1 and S2 Tables). However, we did not observe any significant effect of the index cases’ vaccination status on their contacts’ test results. Second, contacts from cases who were asymptomatic at diagnosis had lower odds of testing positive for the virus (household: aOR: 0.62 [95% CI: 0.53-0.72]; dormitory: aOR: 0.74 [95% CI: 0.58-0.95]) than those from symptomatic cases. Third, contacts exposed during the Omicron period had at least three times higher odds of testing positive (household: aOR: 3.14 [2.54-3.89]; dormitory: aOR: 3.55 [95% CI: 2.17-5.82]).

**Table 3:** Risk factors of SARS-CoV-2 virus transmission among household and dormitory contact members in Brunei Darussalam, August 2021 to February 2022.

		Household contacts			Dormitory contacts		
		n (%)^a^	Crude OR (95% CI)	Adj. OR (95% CI)	n (%)^a^	Crude OR (95% CI)	Adj. OR (95% CI)
Contact characteristics		14243 (100)			10896 (100)		
Gender	Female	6686 (47)	ref		629 (6)	ref	
	Male	7557 (53)	**0.9 (0.84, 0.96)**	0.94 (0.87, 1.01)	10267 (94)	1.28 (0.92, 1.78)	1.24 (0.88, 1.76)
Age group (years)	0-19	5147 (36)	ref		74 (1)	ref	
	20-39	5433 (38)	**0.59 (0.53, 0.64)**	0.95 (0.85, 1.06)	6676 (61)	0.92 (0.49, 1.73)	1.15 (0.58, 2.25)
	40-59	2594 (18)	**0.79 (0.71, 0.88)**	**1.22 (1.08, 1.38)**	4066 (37)	0.86 (0.46, 1.63)	1.12 (0.57, 2.21)
	60+	1069 (8)	**0.73 (0.62, 0.85)**	**1.21 (1.01, 1.45)**	80 (1)	0.64 (0.27, 1.49)	0.65 (0.25, 1.65)
Vaccination status at exposure	None	7799 (55)	ref		3225 (30)	ref	
	One dose	2305 (16)	**0.46 (0.41, 0.53)**	**0.49 (0.43, 0.57)**	2577 (23)	**0.63 (0.51, 0.77)**	**0.67 (0.55, 0.81)**
	Two doses	3445 (24)	**0.55 (0.49, 0.62)**	**0.37 (0.32, 0.43)**	4906 (45)	**0.56 (0.47, 0.70)**	**0.55 (0.42, 0.67)**
	Three doses	694 (5)	0.88 (0.72, 1.08)	**0.30 (0.22, 0.39)**	188 (2)	1.03 (0.63, 1.67)	**0.36 (0.20, 0.64)**
Relationship with index	Immediate family	6303 (44)	ref		119 (1)	ref	
	Extended family	748 (40)	**0.62 (0.55, 0.70)**	**0.63 (0.56, 0.71)**	68 (1)	1.48 (0.60, 3.69)	1.43 (0.53, 3.87)
	Other	1793 (13)	**0.58 (0.49, 0.68)**	**0.60 (0.50, 0.71)**	2567 (24)	1.25 (0.71, 2.20)	1.16 (0.65, 2.08)
	Work/School	399 (3)	**0.22 (0.17, 0.42)**	**0.34 (0.17, 0.65)**	8142 (74)	1.15 (0.67, 1.99)	1.11 (0.62, 1.98)
Case characteristics		2795 (100)			1070 (100)		
Gender	Female	1318 (47)	ref		82 (8)	ref	
	Male	1477 (53)	0.97 (0.85, 1.10)	1.02 (0.90, 1.16)	988 (92)	**1.55 (1.09, 2.22)**	**1.70 (1.14, 2.54)**
Age group (years)	0-19	752 (27)	ref		8 (1)	ref	
	20-39	1153 (41)	1.07 (0.91, 1.25)	1.06 (0.89, 1.26)	684 (64)	1.11 (0.29, 4.24)	1.07 (0.25, 4.58)
	40-59	693 (25)	1.02 (0.85, 1.22)	1.01 (0.83, 1.23)	371 (34)	0.89 (0.23, 3.42)	0.88 (0.20, 3.82)
	60+	197 (7)	0.89 (0.69, 1.15)	0.95 (0.73, 1.25)	7 (1)	**4.60 (1.03, 20.6)**	5.01 (0.98, 26.7)
Vaccinated at diagnosis	No	1674 (60)	ref		389 (36)	ref	
	Yes	1121 (40)	0.99 (0.87, 1.12)	0.88 (0.74, 1.04)	682 (64)	**0.77 (0.60, 0.99)**	0.95 (0.72, 1.26)
Asymptomatic at diagnosis	No	1837 (66)	ref		455 (43)	ref	
	Yes	958 (34)	**0.55 (0.47, 0.63)**	**0.62 (0.53, 0.72)**	615 (57)	**0.65 (0.51, 0.82)**	**0.74 (0.58, 0.95)**
Wave period	Delta	2264 (81)	ref		1013 (95)	ref	
	Omicron	531 (19)	**1.99 (1.72, 2.29)**	**3.14 (2.54, 3.89)**	57 (5)	**2.31 (1.50, 3.56)**	**3.55 (2.17, 5.82)**

CI, confidence interval; OR, odds ratio; ref, reference. The boldface indicates results that were statistically significant.

a Actual counts and proportion included in the GEE analysis, after removing rows with NA values.A total of 146 and 4 rows were removed for household and dormitory clusters, respectively.

Older household contacts had about 1.2 times higher odds of testing positive for SARS-CoV-2 than those aged 0-19 years (40-59 years: aOR: 1.22 [1.08-1.38]; >60 years: aOR: 1.21 [95% CI: 1.01-1.45]). Also, household contacts who were immediate family members had at least 40% higher odds of testing positive for SARS-CoV-2 (extended family: aOR: 0.63 [0.56-0.71]; other: aOR: 0.60 [95% CI: 0.50-0.71]; work/school: aOR: 0.34 [95% CI: 0.17-0.65]). Lastly, dormitory contacts of male index cases had 1.7 times higher odds of testing positive for the virus (aOR: 1.70 [95% CI: 1.14-2.54]).

## Discussion

We assessed the transmission characteristics of SARS-CoV-2 variants in two types of enclosed living settings that constituted 77% of all cases in the study and found four common characteristics. First, our SAR results are higher in the Omicron period, which relates to our higher aOR finding for Omicron. There is a consensus within the scientific community that the Omicron variant is more transmissible than Delta [9,17]. Second, asymptomatic index cases had lower odds of successful onward transmission to their contacts, an intuitive finding that was previously reported for the wild-type and Delta variant [18,19]. Third, a protective effect was observed among vaccinated contacts, regardless of the doses administered; a finding in line with previous reports [20]. Fourth, the SAR was quite similar in both household (31.7%) and dormitory (27.4%) clusters, in contrast with other reports. Studies from the United Kingdom have previously reported lower SAR in larger households [12,21], although their maximum household size was 10. Although a Japanese study reported higher SAR for the dormitory than household clusters, their dormitories were school-based, with a maximum of two residents per private room [7].

Several differences were also observed. First, the median SI was shorter for the household (3 days) than for the dormitory (5 days) cluster types. Additional multivariate GEE analysis tells us that pairs detected during the Omicron period were associated with a 44% decrease in the expected SI (adjusted rate ratio: 0.56 [95% CI: 0.51-0.61]) (S3 Table). Notably, fewer pairs were identified from dormitories during Omicron: 17.8% of all dormitory pairs were from Omicron, compared with 51.7% of all household pairs. This stark difference was related to the 30-day cooling-off period imposed only on dormitories before they can report again as a SARS-CoV-2 case, potentially leading to missed case detection.

Second, our findings for older household contact age and contacts’ relationship with the index case suggest a possible role of housing composition in transmission dynamics. One study reported an association between multi-generational living and severe COVID-19 disease in older South Asian adults [22]. In Brunei, it is common to have multi-generational and extended families living together. In 2021, the average Bruneian household had 5.1 denizens, and 39.4% of all households had a household size of six or more [1]. Notably, we observed 27 households with large household sizes (from 21 to 32), where, upon closer inspection, tells us that they tend to include members from both immediate and extended families. Although all were recorded as living in the same residential address, it could possibly be due to family events occurring at the address. Such gatherings are a usual social activity that could have occurred despite the imposed movement restriction outside the household. Because there is no established national maximum limit to household sizes, we decided not to exclude them from the analysis as possible outliers. However, although such large clusters may not be household members, transmission may have occurred within that address. Taken together, understanding the role of housing composition in within-household transmission in the local context and the role of the household setting as a venue for private gatherings could be helpful to plan targeted public health interventions for future pandemics.

Third, dormitory contacts of male index cases had higher odds of testing positive for SARS-CoV-2. This could be explained by the high proportion of non-local workers employed for labor-intensive industries in Brunei [3].

This study has several limitations. First, we assumed that the index case is the starting point of the infection within each enclosed cluster type, which could introduce uncertainties to our SI and SAR calculations. Infector and infectee misclassification are probable because we relied only on available epidemiologic information. It is also possible for contacts to get infected outside of their enclosed living setting, leading to potential overestimation in our SAR point estimates. However, our SAR results could have already been underestimated because contacts with missing test results were considered SARS-CoV-2–negative, and, in large dormitory clusters, multiple SARS-CoV-2 positive contacts may not be recorded due to operational and time constraints. These points should be considered when interpreting our SI and SAR results. Second, the analysis also relied on the correct determination of cluster types; possible errors made during this manual process could have resulted in under-reported household and/or dormitory clusters. Third, the reported asymptomatic counts do not reflect the proportion of true asymptomatic cases because their symptom status was only determined at diagnosis. Thus, cases deemed asymptomatic in our study could be presymptomatic rather than never-symptomatic. Fourth, there were operational differences in the contact tracing activities conducted in both periods. The sudden increase in case counts during Omicron, coupled with health care worker fatigue, made it infeasible to perform contact tracing with the same rigor as for the Delta period. This also leads to the big differences in the data collection period between Delta (21 weeks) and Omicron (4 weeks) periods. These points should be considered when interpreting our OR results. Fifth, we did not collect information on the housing structure, which could impact disease transmission in enclosed settings. Although the housing layout for a typical household can be quite similar, this may not be true for dormitories, whose building structure, size, and layout could vary drastically depending on their employers’ decisions. General dormitory types range from shophouse flats to housing complexes and could house between two and 200 workers. Dormitory tenants generally stayed in rooms with bunk beds, and shared bathroom and kitchen facilities. Although mainly reserved for workers, there were also rare instances where children stayed with their working parents in dormitories. These points, along with composition differences between households (typically, immediate family members) and dormitories (mainly, foreign workers), should be considered when assessing the comparability of both settings. Sixth, we did not consider the impact of physical distancing and infection control measures possibly taken by cases and contacts within their living settings. Lastly, we also did not consider the co-morbidity status and COVID-19 disease severity for the cases.

Our study findings provide country- and setting-specific insights into the transmission dynamics of the SARS-CoV-2 virus, which could help guide future outbreak investigations for the next novel respiratory disease and refine disaster management policies at the national level. One lesson learned from the pandemic is the need to adopt the Health in All Policies approach [23], with involvement from ministries and sectors other than health. From this perspective, our study findings could provide some evidence across all sectors toward promoting the health of all local and non-local residents in the country.

## Conclusion

We report substantial onward transmission of SARS-CoV-2 Delta and Omicron variants within enclosed living settings and that factors associated with onward transmission differ between them, particularly, in terms of the age and relationship among household members. Further research on how housing composition impacts within-household transmission in the context of an Asian-Pacific society is warranted.

## Declaration of competing interest

The authors have no competing interests to declare.

## Appendix Supplementary materials

Image, application 1
