# Supplementary material for: Factors associated with onward SARS-CoV-2 transmission among household and dormitory contacts of cases in Brunei Darussalam, August 2021 to February 2022: A retrospective cohort study
Source: IJID Reg. 2026 Apr 1;19:100888. doi: 10.1016/j.ijregi.2026.100888 (PMC13094499; doi:10.1016/j.ijregi.2026.100888)
Supplement: Supplementary file 1 [file mmc1.docx]

**Supplementary Information file** for study entitled “Factors associated with onward SARS-CoV-2 transmission among household and dormitory contacts of cases in Brunei Darussalam, August 2021 – February 2022: A retrospective cohort study”

**S1 Text**

**Case identification and management**

A suspected SAR-CoV-2 case is one meeting any of the following criteria: (i) Contacts of a confirmed case (regardless of symptoms); (ii) Arrived in the country as a traveller or returning resident, and (iii) Admitted to an inpatient facility with pneumonia.

All confirmed cases were initially transported, isolated, and treated at the National Isolation Centre. However, from September 2021, confirmed COVID-19 cases were allowed to undergo home isolation if their symptoms were mild and their homes were deemed appropriate for isolation. They were assessed daily by trained personnel for clinical deterioration and welfare via telephone. From January 2022, COVID-19 isolation requirements were again relaxed by considering the vaccination status of confirmed cases; fully vaccinated and unvaccinated cases were required to isolate for 10 and 14 days, respectively.

**Contact identification and management**

Following notification of a confirmed case, a contact tracer was tasked to inform each case of the diagnosis and gather the following information from them: Demographics, presenting symptoms, travel history, and movement activities at least 2 days before symptom onset date (or swab date for asymptomatic cases). Information on presenting symptoms was collected once during history taking or swab collection. It included the following: fever, cough, sore throat, runny nose, sneeze, shiver, shortness of breath, headache, body and joint pain, body weakness, nausea, vomiting, and loss of taste, smell, and appetite.

All case information collected was cross-checked with existing data from the centralized national electronic patient records database. Upon receiving a positive notification, it would be assigned to a contact tracer who would contact the case immediately and get their identified contacts tested. Any logistics issues would be expedited. Most contacts got tested within the same day or the day after identification, depending on notification timing; the average turnaround time for identifying and testing contacts was 2 days. Epidemiologic and clinical information from the identified contacts was input by the contact tracer into a standardised and computerised form and saved in a dedicated database.

All identified close contacts from contact tracing efforts were contacted and scheduled for RT-PCR testing, irrespective of symptom presentation. Those who tested negative were quarantined at home for 14 days from the date of last exposure. Close contacts were released from quarantine on day 15 post-exposure, if they remained asymptomatic or continued to test negative for SARS-CoV-2 virus. Those who developed symptoms during quarantine were retested. From January 2022, quarantine requirements were also relaxed for contacts, whereby vaccinated and unvaccinated close contacts were required to quarantine for 5 and 10 days, respectively. In cases of home quarantine, close contacts were assessed daily by trained personnel for clinical deterioration and welfare via telephone.

**Cluster identification method**

Cluster identification was conducted only in the case dataset. Between May and December 2022, a group of contact tracers previously involved in contact tracing work were tasked to retrospectively identify potential enclosed setting clusters from the COVID-19 case list, based on the residential address, symptom onset/swab collection dates & observations during case detection. Cluster types were initially classified into four categories: (a) Household defined as occurring within a typical household; (b) Dormitory within accommodation for workers; (c) Institution within a defined enclosed establishment within one or a group of separate buildings (such as prison or rehabilitation centre), and (d) Workplace within a defined workplace (such as offices, petroleum offshore platforms and military camps). A combination of variables was used when classifying each cluster into 1 of the 4 cluster types: original “operational” cluster name, case number of the possible infection source, and home type (private residence, dormitory, staff house, military camps, etc.). These variables were already collected as part of the outbreak investigation. Upon completion of this cluster identification step, the resulting cluster list was further cleaned to meet the study’s cluster definition, that is, two or more epidemiologically linked cases. Further analyses in the study focused only on the household and dormitory cluster types, due to the low counts for the institution and workplace.

Though the above-outlined process was manual and tedious, it was necessary as the clusters identified during the outbreak period were based on the initial point of detection. For example, if an outbreak was detected in a school, all identified SARS-CoV-2 cases and their contacts (both school and household) were categorised as the school cluster. During the pandemic period, operational cluster names were created during the contact tracing process to quickly identify cluster names based on the original location where a case was detected, such as “Restaurant A”, “School QRS”, and “XYZ Construction staffhouse”. For household-based clusters, the cluster was named using the case number of the first case detected, for example, in “Cluster 10890”, the first case detected (and therefore assuming infection source for that cluster) was case no. 10890.

**Serial Interval (SI) pair determination**

The SI is defined as the difference between the symptom onset dates of the infector and that of the infectee. Within each cluster, we first identify the infector, defined as one with the earliest symptom onset and with the smallest case number (these were assigned as running numbers based on the date and time of notification received by the outbreak response team; hence, smaller case numbers meant earlier notification).

**COVID-19 vaccination implementation in Brunei**

Both AstraZeneca and Sinopharm vaccines were introduced into the population in the initial stages of the pandemic (first half of 2021), followed by RNA vaccines (Moderna and Pfizer) from the second half of 2021 to mid-2022. Initial vaccination rollout was carried out in three phases: (1) Frontline workers, adults ≥ 60 years and students bound for overseas study, (2) Teachers, childcare workers and adults with comorbidities, (3) All adults ≥ 18 years. Vaccine rollout to those between 12 and 17 years began in early December 2021, and then to subsequent younger ages from the first half of 2022. While vaccination uptake was not made mandatory, measures were imposed such that “daily living was easier” if one was vaccinated with two doses: Access to indoor public and business premises was given only to those vaccinated with two doses (through use of vaccination passport functionality in the then contact tracing mobile application, BruHealth [1]), and government employees who were not vaccinated with two doses have to undergo rapid antigen testing twice a week before going to their workplace, from November 2021 to March/April 2022.

**S1 Fig.** Timeline of public health interventions and COVID-19 vaccination implementation in Brunei Darussalam, March 2021 to April 2022. The area shaded in green shows the overall study period.


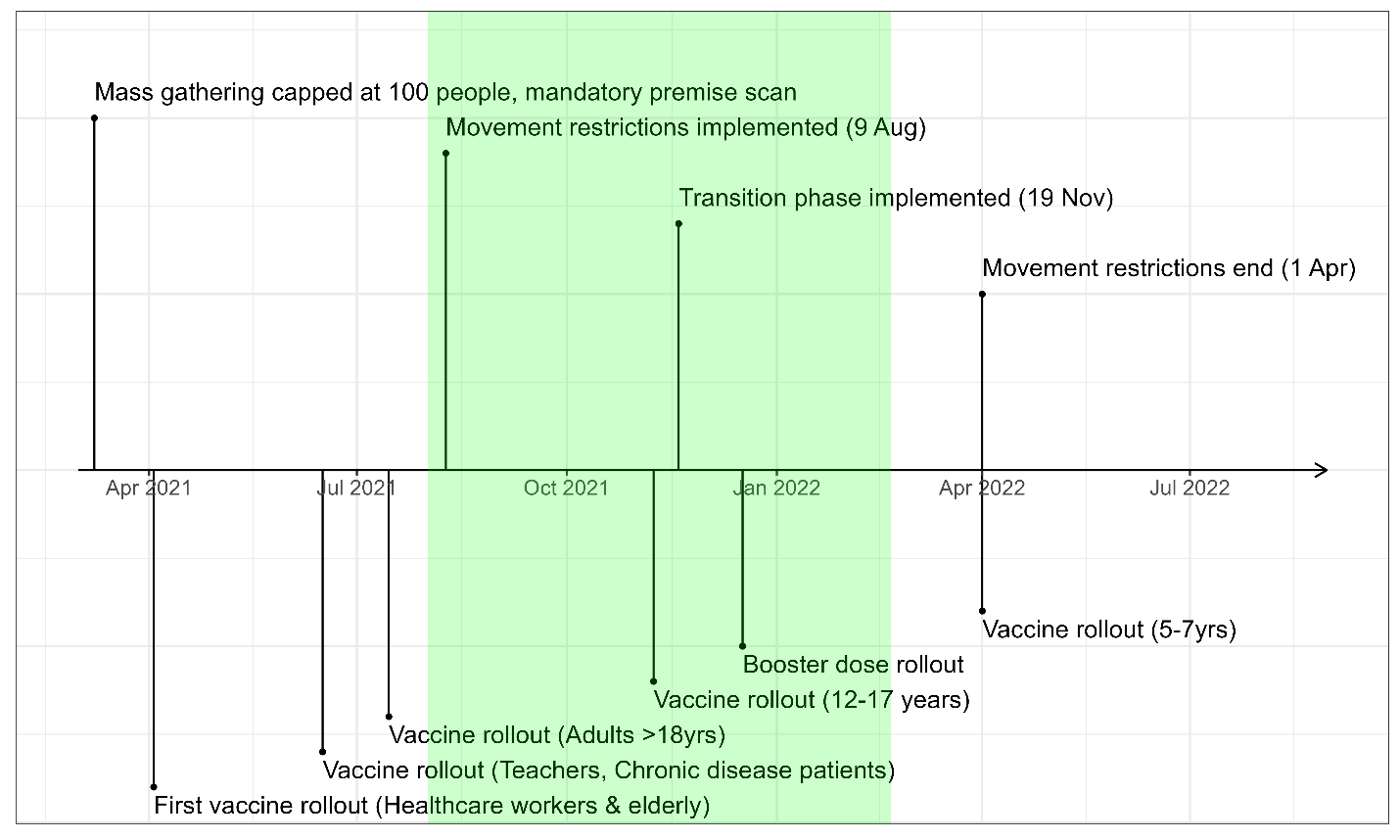


The study period (in green) covers all four phases of the national COVID-19 Recovery Framework, which was initiated in response to the Delta wave [2]. The four phases are: Containment (7th Aug – 3rd Oct 2021), Preparation (4th Oct – 18th Nov 2021), Transition (19th Nov – 14th Dec 2021), and Endemic (from 15th Dec 2021 onwards). Movement restrictions ended on 1st Apr 2022.

**S2 Fig.** Epidemic curve of COVID-19 cases in Brunei Darussalam, August 2021 to May 2022. The area shaded in blue shows the variant switch period, acting as a divider between the Delta and Omicron periods. The area shaded in orange shows case counts outside the study period; this is included here to show the full Omicron wave.


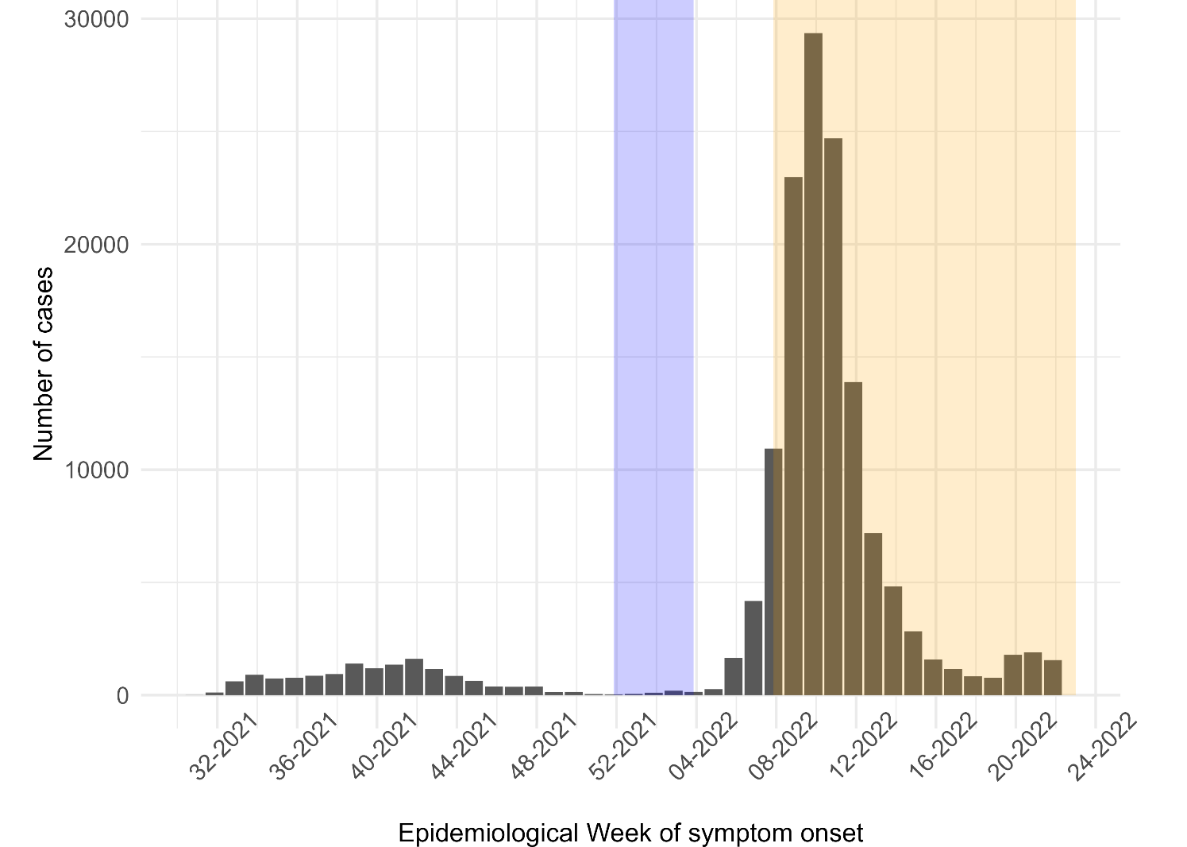


**S3 Fig.** Histogram showing the number of Delta and Omicron variants sequenced from a selected proportion of case samples, plotted by the epidemiological week of symptom onset (or swab collection) of the case.


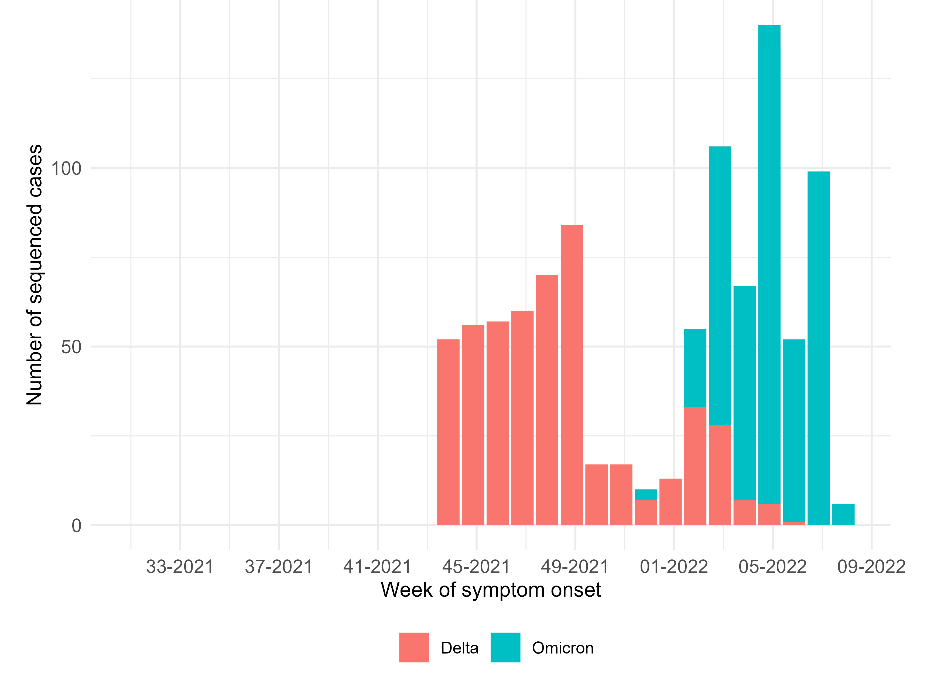


**S4 Fig.** Flowchart showing the breakdown and exclusion rationale of contact inclusion into the study. Contacts included in the regression analyses are indicated as grey-shaded boxes.


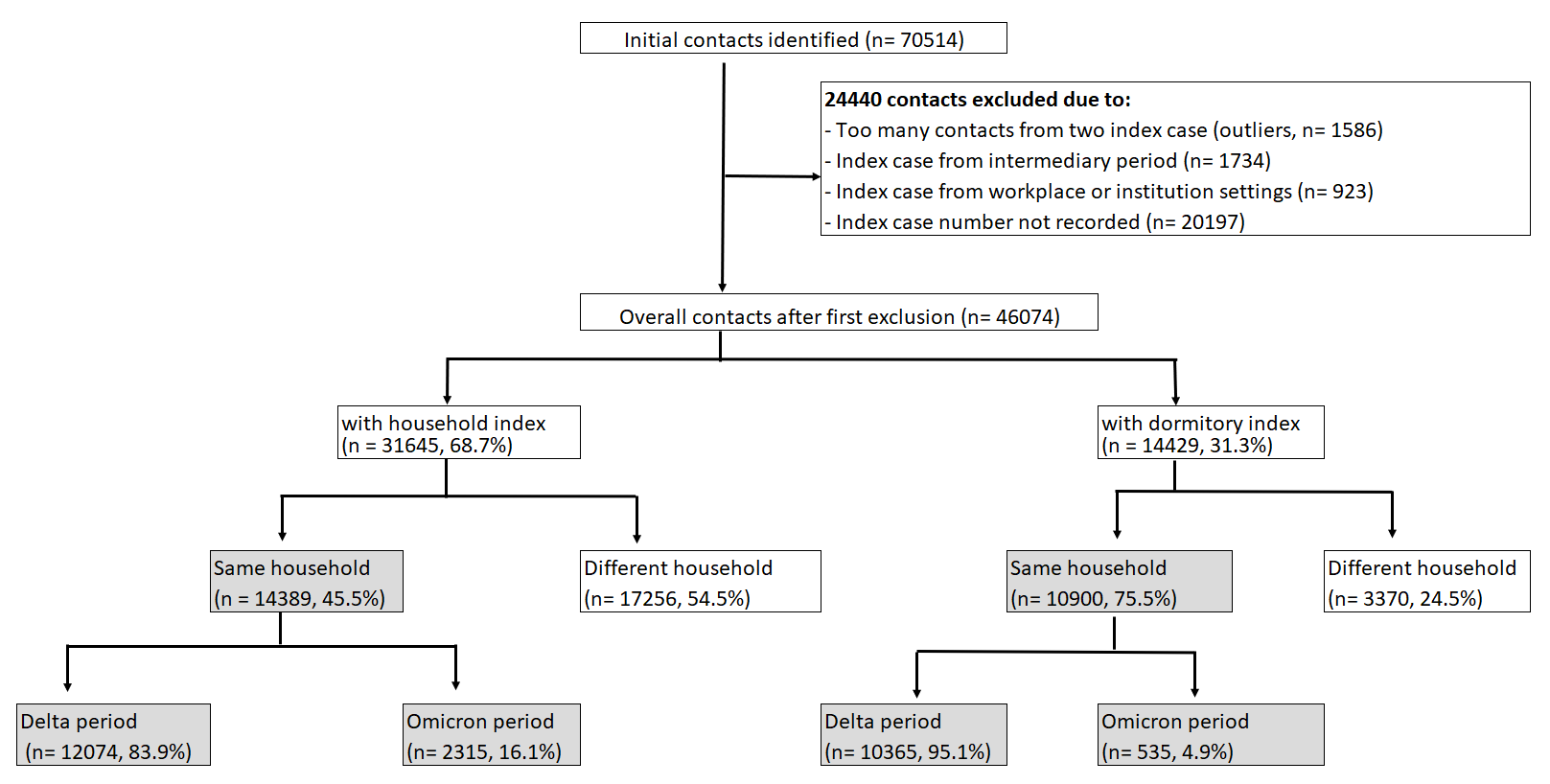


**S1 Table.** Risk factors of SARS-CoV-2 virus transmission among household and dormitory contact members in Brunei Darussalam during the Delta period, August – December 2021

|  | | Household contacts | | | Dormitory contacts | | |
| --- | --- | --- | --- | --- | --- | --- | --- |
|  |  | n (%)* | Crude OR (95% CI) | Adj. OR (95% CI) | n (%)* | Crude OR (95% CI) | Adj. OR (95% CI) |
| Contact characteristics | | 11937 (100) | | | 10361 (100) | | |
| Gender | Female | 5487 (46) | ref |  | 538 (5) | ref |  |
|  | Male | 6450 (54) | **0.9 (0.83, 0.97)** | 0.92 (0.85, 1.00) | 9823 (95) | **1.43 (1.11, 1.84)** | 1.28 (0.99, 1.66) |
| Age group (years) | 0-19 | 4314 (36) | ref |  | 65 (1) | ref |  |
|  | 20-39 | 4603 (39) | **0.59 (0.53, 0.66)** | 0.95 (0.84, 1.07) | 6398 (62) | 1.14 (0.53, 2.47) | 1.29 (0.55, 3.03) |
|  | 40-59 | 2144 (18) | **0.77 (0.68, 0.87)** | **1.20 (1.05, 1.38)** | 3823 (36) | 1.05 (0.49, 2.28) | 1.26 (0.53, 2.97) |
|  | 60+ | 876 (7) | **0.70 (0.59, 0.84)** | 1.22 (1.00, 1.49) | 75 (1) | 0.77 (0.28, 2.11) | 0.68 (0.23, 2.02) |
| Vaccination status at exposure | None | 7268 (61) | ref |  | 3206 (31) | ref |  |
|  | One dose | 2298 (19) | **0.49 (0.43, 0.56)** | **0.47 (0.41, 0.55)** | 2576 (25) | **0.63 (0.52, 0.76)** | **0.66 (0.54, 0.79)** |
|  | Two doses | 2319 (19) | **0.31 (0.27, 0.36)** | **0.31 (0.26, 0.36)** | 4568 (44) | **0.50 (0.42, 0.60)** | **0.53 (0.44, 0.64)** |
|  | Three doses | 52 (1) | 0.07 (0.02, 0.29) | **0.08 (0.02, 0.32)** | 11 (0) | 0.18 (0.02, 1.45) | 0.15 (0.01, 1.54) |
| Relationship with index | Immediate family | 5066 (43) | ref |  | 100 (1) | ref |  |
|  | Extended family | 5050 (42) | **0.66 (0.58, 0.74)** | **0.64 (0.57, 0.72)** | 51 (0) | 2.98 (1.24, 7.18) | 3.08 (1.18, 8.04) |
|  | Other | 1484 (12) | **0.59 (0.50, 0.69)** | **0.60 (0.50, 0.71)** | 2462 (24) | 1.61 (0.91, 2.85) | 1.19 (0.61, 2.33) |
|  | Work/School | 337 (3) | **0.24 (0.15, 0.37)** | **0.38 (0.23, 0.62)** | 7748 (75) | 1.52 (0.86, 2.66) | 1.17 (0.60, 2.28) |
| Case characteristics | | 2264 (100) | | | 1013 (100) | | |
| Gender | Female | 1051 (46) | ref |  | 66 (7) | ref |  |
|  | Male | 1213 (54) | 0.98 (0.86, 1.10) | 1.03 (0.91, 1.17) | 947 (93) | **1.85 (1.34, 2.55)** | **1.69 (1.20, 2.38)** |
| Age group (years) | 0-19 | 611 (27) | ref |  | 6 (1) | ref |  |
|  | 20-39 | 925 (41) | 1.16 (1.00, 1.36) | 1.05 (0.96, 1.35) | 655 (64) | 1.66 (0.62, 4.44) | 1.94 (0.59, 6.36) |
|  | 40-59 | 572 (25) | 1.10 (0.93, 1.31) | 1.20 (0.88, 1.26) | 345 (34) | 1.33 (0.50, 3.57) | 1.66 (0.50, 5.47) |
|  | 60+ | 156 (7) | 0.96 (0.75, 1.22) | 1.22 (0.76, 1.29) | 7 (1) | **7.23 (2.35, 22.3)** | **9.83 (2.60, 37.1)** |
| Vaccinated at diagnosis | No | 1598 (71) | ref |  | 385 (38) | ref |  |
|  | Yes | 666 (29) | 0.76 (0.67, 0.86) | 0.95 (0.82, 1.10) | 628 (62) | **0.71 (0.61, 0.83)** | 0.97 (0.82, 1.13) |
| Asymptomatic at diagnosis | No | 1393 (61) | ref |  | 416 (41) | ref |  |
|  | Yes | 871 (39) | **0.58 (0.51, 0.66)** | **0.61 (0.54, 0.70)** | 597 (59) | **0.69 (0.60, 0.80)** | **0.74 (0.64, 0.86)** |

*Actual counts and proportions included in the GEE analysis, after removing rows with NA values.

A total of 137 and 4 rows were removed for household and dormitory clusters, respectively.

**S2 Table.** Risk factors of SARS-CoV-2 virus transmission among household and dormitory contact members in Brunei Darussalam during the Omicron period, January – February 2022

|  | | Household contacts | | | Dormitory contacts | | |
| --- | --- | --- | --- | --- | --- | --- | --- |
|  |  | n (%)* | Crude OR (95% CI) | Adj. OR (95% CI) | n (%)* | Crude OR (95% CI) | Adj. OR (95% CI) |
| Contact characteristics | | 2306 (100) | | | 535 (100) | | |
| Gender | Female | 1199 (52) | ref |  | 91 (17) | ref |  |
|  | Male | 1107 (48) | 0.99 (0.85, 1.16) | 1.00 (0.84, 1.18) | 444 (83) | 1.41 (0.75, 2.64) | 0.83 (0.49, 1.41) |
| Age group (years) | 0-19 | 833 (36) | ref |  | 9 (2) | ref |  |
|  | 20-39 | 830 (36) | **0.58 (0.47, 0.72)** | 0.83 (0.62, 1.12) | 278 (52) | 0.41 (0.10, 1.71) | 0.20 (0.03, 1.40) |
|  | 40-59 | 450 (20) | 0.83 (0.65, 1.06) | 1.19 (0.89, 1.59) | 243 (45) | 0.41 (0.12, 1.37) | 0.19 (0.03, 1.37) |
|  | 60+ | 193 (8) | 0.76 (0.54, 1.08) | 1.14 (0.75, 1.71) | 5 (1) | 0.33 (0.04, 3.06) | 0.15 (0.01, 2.53) |
| Vaccination status at exposure | None | 531 (23) | ref |  | 19 (4) | ref |  |
|  | One dose | 7 (0) | 0.82 (0.21, 3.17) | 0.79 (0.21, 3.00) | 1 (0) | 3.50 (0.28, 43.3) | 9.88 (0.23, 423.2) |
|  | Two doses | 1126 (49) | **0.66 (0.52, 0.84)** | **0.71 (0.52, 0.98)** | 338 (63) | 3.60 (0.77, 17.0) | **6.08 (1.39, 26.6)** |
|  | Three doses | 642 (28) | **0.48 (0.36, 0.66)** | **0.49 (0.32, 0.74)** | 177 (33) | 2.09 (0.58, 7.45) | **3.86 (1.07, 13.9)** |
| Relationship with index | Immediate family | 1237 (54) | ref |  | 19 (4) | ref |  |
|  | Extended family | 698 (30) | **0.63 (0.50, 0.79)** | **0.61 (0.48, 0.77)** | 17 (3) | 0.06 (0.01, 0.46) | **0.08 (0.01, 0.81)** |
|  | Other | 309 (13) | **0.56 (0.40, 0.79)** | **0.55 (0.39, 0.78)** | 105 (20) | 1.25 (0.40, 3.94) | **1.32 (0.37, 4.74)** |
|  | Work/School | 62 (3) | **0.18 (0.04, 0.90)** | **0.18 (0.04, 0.77)** | 394 (74) | 0.69 (0.20, 2.33) | **0.78 (0.23, 2.66)** |
| Case characteristics | | 531 (100) | | | 57 (100) | | |
| Gender | Female | 267 (50) | ref |  | 16 (28) | ref |  |
|  | Male | 264 (50) | 0.98 (0.77, 1.25) | 0.99 (0.77, 1.27) | 41 (72) | **2.17 (1.12, 4.19)** | **2.02 (1.10, 3.69)** |
| Age group (years) | 0-19 | 141 (26) | ref |  | 2 (3) | - |  |
|  | 20-39 | 228 (43) | 0.67 (0.51, 0.89) | 0.84 (0.56, 1.24) | 29 (51) | 1.96 (0.99, 3.86) |  |
|  | 40-59 | 121 (23) | 0.77 (0.53, 1.12) | 0.88 (0.55, 1.41) | 26 (46) | ref |  |
|  | 60+ | 41 (8) | 0.60 (0.39, 0.94) | 0.77 (0.46, 1.31) | 0 (0) | - |  |
| Vaccinated at diagnosis | No | 76 (14) | ref |  | 4 (7) | ref |  |
|  | Yes | 455 (86) | **0.58 (0.42, 0.79)** | 0.62 (0.38, 1.00) | 53 (93) | 0.41 (0.06, 2.70) | 0.27 (0.05, 1.60) |
| Asymptomatic at diagnosis | No | 444 (84) | ref |  | 39 (68) | ref |  |
|  | Yes | 87 (16) | **0.66 (0.47, 0.94)** | **0.64 (0.45, 0.91)** | 18 (32) | 0.53 (0.27, 1.04) | 0.64 (0.36, 1.15) |

*Actual counts and proportions included in the GEE analysis, after removing rows with NA values.

A total of 6 and 0 rows were removed for household and dormitory clusters, respectively.

**S3 Table.** Association between infector and infectee characteristics (independent variables) and SI (dependent variable)

| Independent variables | | *n (%) | Crude RR (95% CI) | Adj. RR (95% CI) |
| --- | --- | --- | --- | --- |
| Unique infector characteristics (N = 605) | | | |  |
| Gender | Female | 262 (43) | 1 | 1 |
|  | Male | 343 (57) | 1.07 (0.99, 1.16) | 1.02 (0.95, 1.09) |
| Age group | 0-19 | 167 (28) | 1 | 1 |
|  | 20-39 | 256 (42) | **1.18 (1.07, 1.30)** | **1.16 (1.05, 1.28)** |
|  | 40-59 | 148 (24) | **1.12 (1.01, 1.25)** | 1.07 (0.96, 1.18) |
|  | 60+ | 34 (6) | 1.13 (0.93, 1.38) | 1.09 (0.91, 1.30) |
| Vaccinated at infection | No | 271 (45) | 1 | 1 |
|  | Yes | 335 (55) | **0.72 (0.67, 0.77)** | 0.93 (0.85, 1.02) |
| Infectee characteristics (N = 3787) | | |  |  |
| Gender | Female | 1700 (45) | 1 | 1 |
|  | Male | 2087 (55) | 1.05 (1.00, 1.11) | **1.06 (1.02, 1.10)** |
| Age group | 0-19 | 1296 (34) | 1 | 1 |
|  | 20-39 | 1497 (40) | 0.99 (0.92, 1.05) | 0.98 (0.91, 1.06) |
|  | 40-59 | 805 (21) | 0.99 (0.91, 1.07) | 0.96 (0.90, 1.03) |
|  | 60+ | 189 (5) | 0.99 (0.88, 1.11) | 0.97 (0.87, 1.09) |
| Vaccinated at infection | No | 1882 (50) | 1 | 1 |
|  | Yes | 1905 (50) | 1.10 (0.98, 1.24) | 1.09 (0.99, 1.19) |
| Cluster type | Dormitory | 757 (20) | 1 | 1 |
|  | Household | 3030 (80) | **0.75 (0.67, 0.84)** | 0.93 (0.83, 1.05) |
| Wave period | Delta | 2085 (55) | 1 | 1 |
|  | Omicron | 1702 (45) | **0.55 (0.51, 0.58)** | **0.56 (0.51, 0.61)** |

GEE models were used with numeric SI as the outcome variable (numeric SI value). Due to SI’s right-skewed distribution, a gamma distribution with a log link function was used. Clustering was accounted for, and an exchangeable working correlation was used.

*Actual counts and proportions included in the GEE analysis, after removing rows with NA values.

References:

1. Koh KS, Lim HS, Lim J, Wei Y, Minn PW, Wong J. Development and implementation of a national mobile health application: A case study from Brunei. J Glob Health. 2022;12:1–5.

2. Prime Minister’s Office Brunei Darussalam. National COVID-19 Recovery Framework. 2021. https://www.pmo.gov.bn/SiteCollectionDocuments/covid19/National-COVID-19-Recovery-Framework.pdf. Accessed 22 Oct 2024.
